# Transcription Factors as Therapeutic Targets in Chronic Kidney Disease

**DOI:** 10.3390/molecules23051123

**Published:** 2018-05-09

**Authors:** Akihito Hishikawa, Kaori Hayashi, Hiroshi Itoh

**Affiliations:** Division of Nephrology, Endocrinology and Metabolism, Department of Internal Medicine, Keio University School of Medicine, Tokyo 160-8582, Japan; a-hishikawa@keio.jp (A.H.); hiito@keio.jp (H.I.)

**Keywords:** chronic kidney disease (CKD), Nrf2, bardoxolone methyl, HIF, KLF4, renin–angiotensin system (RAS)

## Abstract

The growing number of patients with chronic kidney disease (CKD) is recognized as an emerging problem worldwide. Recent studies have indicated that deregulation of transcription factors is associated with the onset or progression of kidney disease. Several clinical trials indicated that regression of CKD may be feasible via activation of the transcription factor nuclear factor erythroid-2 related factor 2 (Nrf2), which suggests that transcription factors may be potential drug targets for CKD. Agents stabilizing hypoxia-inducible factor (HIF), which may be beneficial for renal anemia and renal protection, are also now under clinical trial. Recently, we have reported that the transcription factor Kruppel-like factor 4 (KLF4) regulates the glomerular podocyte epigenome, and that the antiproteinuric effect of the renin–angiotensin system blockade may be partially mediated by KLF4. KLF4 is one of the Yamanaka factors that induces iPS cells and is reported to be involved in epigenetic remodeling. In this article, we summarize the transcription factors associated with CKD and particularly focus on the possibility of transcription factors being novel drug targets for CKD through epigenetic modulation.

## 1. Introduction

Chronic kidney disease (CKD) is now a global health burden, and its prevalence is estimated at more than 10%, corresponding to almost 500 million people around the world [1,2,3,4]. Though CKD is usually asymptomatic until the later stages, all stages of CKD are associated with increased risk of cardiovascular disease [5]; thus, treatment for CKD is a major research issue. However, there is no specific treatment for CKD at present. The current treatment mainly focuses on blood pressure management using renin–angiotensin system (RAS) inhibitors (angiotensin-converting enzyme inhibitors (ACE-I) or angiotensin receptor blockers (ARB)), which may ameliorate proteinuria and decrease the rate of progression to end-stage renal disease. As it has been clarified that oxidative stress, inflammation, and hypoxia contribute to CKD progression [6,7], more specific treatments are being developed to act on such pathways, especially focusing on transcription factors. In this article, we review the two major transcriptional factors, Nrf2 and HIF, and agents targeting them as promising therapies for CKD and reconsider the mechanism of RAS inhibitors, focusing on the transcription factor KLF4 based on our recent work.

## 2. Nuclear Factor Erythroid-2 Related Factor 2 (Nrf2)

### 2.1. Nrf2–Keap1 Antioxidant Pathway

Oxidative stress and inflammation have been suggested as key pathologic components in CKD. Nuclear factor erythroid-2 related factor 2 (Nrf2) is a transcription factor working as a crucial regulator of the antioxidant defense system. While it had been mainly studied in the fields of cancer biology since its discovery in 1994, investigation of its role in non-neoplastic diseases including kidney diseases has begun recently. Several studies have shown that impaired Nrf2 activity is implicated with cardiovascular disease [8] and neurodegenerative diseases [9].

**Figure 1:**
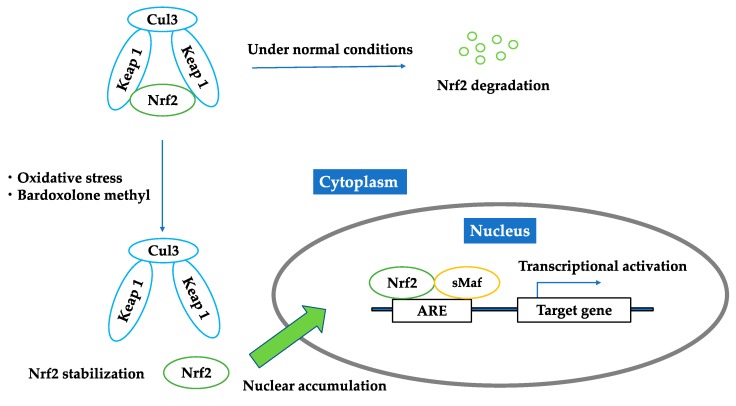
Regulation of Nrf2 under unstressed condition; oxidative stress; bardoxolone methyl. Under normal conditions, Nrf2 is kept in the cytoplasm and continuously degraded by Keap1. Under oxidative stress conditions or the existence of bardoxolone methyl, Nrf2 is released from Keap1. The released Nrf2 translocates into the nucleus, forms heterodimers with other transcription factors, such as small Maf proteins, and binds to the antioxidant response elements and activates downstream genes.

Nrf2 is negatively regulated by Kelch-like ECH-associated protein 1 (Keap1). Keap1 is a cytosolic protein, an adaptor component of the Cul3-based E3 ligase complex, which promotes ubiquitination and degradation of Nrf2 [10]. Thus, under normal conditions, Nrf2 is kept in the cytoplasm and continuously degraded by Keap1. Under oxidative stress conditions, oxidization or covalent modification occurs in the cysteine residues of Keap1, and then Nrf2 is released from Keap1. The released Nrf2 translocates into the nucleus, forms heterodimers with other transcription factors, such as small Maf proteins, and binds to the antioxidant response element (ARE) located in the promoter region of target genes. Nrf2 upregulates hundreds of cytoprotective genes, including antioxidants and phase II detoxifying enzymes such as catalase, superoxide dismutase, heme oxygenase-1 (HO-1), NAD(P)H:quinone oxidoreductase 1 (NQO1), glutathione peroxidase-2, and glutathione S-transferase (Figure 1).

Since Nrf2 is protective against oxidative stress and inflammation, it is natural to believe that its activity is increased in CKD, considering its pathophysiology. In fact, Nrf2 is activated as an adaptive defense against oxidative stress during cholesterol crystal-induced inflammation in macrophages [11]. However, several animal experiment studies have shown that in diseased kidneys, Nrf2 activity and the expression of its target genes were, paradoxically, decreased. Studies with a CKD model using 5/6 nephrectomy rats [12], a spontaneous focal segmental sclerosis model using Imai rats [13], and a tubulointerstitial nephropathy model using adenine treatment [14] showed impaired activity of Nrf2. CKD model using 5/6 nephrectomy rats also implied the negative correlation between the change in Nrf2 activity and CKD progression, since Nrf2 activity in the remnant kidney was mildly reduced at six weeks after the nephrectomy and markedly reduced at 12 weeks after nephrectomy [12]. However, Nrf2 activity in CKD continues to be controversial, partly because the consequence of Nrf2 activation may vary by organ, cell type, and pathology. The precise mechanism of the impaired Nrf2 activity in the kidneys of these animal models were unclear, while indoxyl sulfate, one of the uremic toxins, showed downregulation of renal Nrf2 expression in CKD rats through activation of NF-κβ [15]. Impaired Nrf2 activity through NF-κβ activation has been demonstrated in other chronic inflammatory disorders such as chronic granulomatous disease [16], suggesting that impaired Nrf2 activity in CKD appears to be at least, in part, due to the accompanying systemic inflammation. Moreover, Nrf2-knockout mice developed lupus-like autoimmune nephritis [17] and were more susceptible to hyperglycemia-induced renal injury [18], ischemia reperfusion injury, and cisplatin-induced nephrotoxicity [19] than wild-type mice.

### 2.2. Bardoxolone Methyl as a Nrf2 Activator

**Figure 2:**
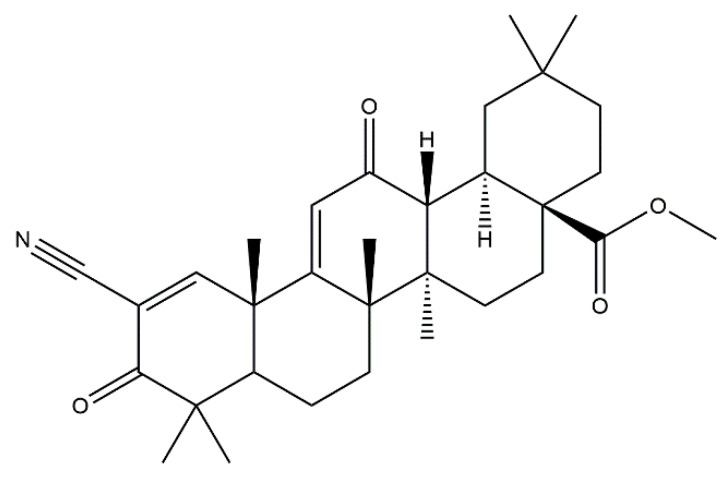
The chemical structure of bardoxolone methyl.

The reports mentioned above imply that the paradoxically impaired Nrf2 activity in kidney is related with the pathophysiology of CKD. Conversely, a renal protective role of Nrf2 is supported by the finding that dietary Nrf2 activators such as sulforaphane or cinnamic aldehyde limited albuminuria and protected against renal oxidative damage in rats with streptozotocin-induced diabetes, suggesting that Nrf2 may be a potent therapeutic target in kidney diseases [20]. Bardoxolone methyl is a semisynthetic triterpenoid, derived from the natural product oleanolic acid, and is known to be one of the most potent inducers of Nrf2 [21,22,23] (Figure 2). Bardoxolone methyl binds to Keap1 and inhibits the ubiquitination activity of Keap1, allowing Nrf2 to translocate to the nucleus and upregulate antioxidant and cytoprotective genes (Figure 1). In addition, bardoxolone methyl exhibits anti-inflammatory activity by inhibiting the IKKβ/NF-κβ signaling pathway [24]. Bardoxolone methyl was originally used in a phase 1 clinical trial as an antioxidant inflammation modulator for advanced solid tumors and lymphomas [25]. During the trial, an increase in the estimated glomerular filtration rate (eGFR) was found by chance with the use of bardoxolone methyl, suggesting that bardoxolone methyl might be beneficial in chronic kidney disease. Moreover, the renoprotective activity of bardoxolone methyl and related analogs have been reported in animal models such as the amelioration of ischemia-reperfusion injuries [26] and streptozotocin-induced diabetic kidney injuries [27]. After the trial mentioned above, bardoxolone methyl was applied in phase 2 clinical trials for patients with diabetes and CKD [28,29]. Diabetic patients were good candidates for treatment with bardoxolone methyl, since the relationship between inflammation and the progression of diabetic nephropathy has been noted [30,31].

At first, an exploratory multicenter, open-label phase 2a clinical study was carried out to assess the activity and safety of bardoxolone methyl [28]. Twenty patients with moderate to severe CKD (eGFR 15–45 mL/min/1.73 m^2^) and type 2 diabetes were enrolled, and they received 25 mg of bardoxolone methyl daily for 28 days, followed by 75 mg daily for another 28 days. Administration of bardoxolone showed a significant increase in the eGFR (7.2 mL/min/1.73 m^2^ at 8 weeks), a decrease in serum creatinine and blood urea nitrogen, and an increase in creatinine clearance without life-threatening adverse events or serious drug-related adverse events. 

Based on the results of the phase 2a clinical trial, a large phase 2b clinical trial (52-Week Bardoxolone Methyl Treatment: Renal Function in CKD/Type 2 Diabetes (BEAM) study) was carried out to assess the longer-term effects and dose response of bardoxolone methyl in CKD patients with type 2 diabetes [29]. The study was a double-blind, randomized, placebo-controlled trial, and 227 CKD patients (eGFR 20–40 mL/min/1.73 m^2^) with type 2 diabetes were enrolled. They received placebo or bardoxolone methyl at a target dose of 25, 75 or 150 mg once daily. Bardoxolone methyl improved the eGFR in a dose-dependent manner at 24 weeks, and these improvements persisted for 52 weeks. Adverse events, including muscle spasms, hypomagnesemia, alanine aminotransferase levels, and gastrointestinal effects, were mild to moderate and manageable.

Given the results of the BEAM study, a phase 3 clinical trial (Bardoxolone Methyl Evaluation in Patients With Chronic Kidney Disease and Type 2 Diabetes: the Occurrence of Renal Events (BEACON) study) was performed to assess whether long-term administration of bardoxolone methyl on a background of standard therapy including RAS inhibitors reduces renal and cardiac morbidity and mortality [32]. The study was a multinational, multicenter, double-blind, randomized, placebo-controlled trial, and 2185 CKD patients (eGFR 15–30 mL/min/1.73 m^2^) with type 2 diabetes were enrolled. The trial would have been the first event-driven trial to evaluate the effect of an oral antioxidant and anti-inflammatory drug in advanced CKD. However, the trial was terminated prematurely based on the recommendation of the Independent Data Monitoring Committee for safety concerns due to excess serious adverse events and mortality. The preliminary analyses revealed that patients with bardoxolone methyl showed significantly higher rates of heart failure events. The additional analysis suggested that bardoxolone methyl may pharmacologically promote acute sodium and volume retention and increase blood pressure due to the suppression of the endothelin pathway, likely through suppression of the NF-κβ pathway [33]. On the other hand, post-hoc analysis suggested that patients randomized to bardoxolone methyl experienced increases in eGFR consistent with prior studies [34]. Although, there are criticisms to the effect on the eGFR which could have been mediated by a potential afferent arteriolar dilatation and increasing intraglomerular pressure, since the chemical structure of bardoxolone methyl is similar to that of cyclopentenone prostaglandins causing renal vasodilatation [35,36]. Hyperfiltration may contribute to renal function loss and progression of nephropathy in the long term [37].

### 2.3. Brief Summary of Targeting Nrf2

Bardoxolone methyl was an epoch-making drug, since the conventional CKD therapy with RAS inhibitors merely delayed its progression, though bardoxolone methyl improved kidney function. The result of the BEACON study was disappointing, but there still remains a possibility that bardoxolone methyl can be used to manage CKD by excluding high-risk heart failure patients. There is a randomized, double-blind, placebo-controlled, multicenter phase 2 trial in Japan (TSUBAKI trial) enrolling 120 patients with CKD and type 2 diabetes without identified risk factors for fluid overload, such as baseline brain natriuretic peptide (BNP) >200 pg/mL and prior history of heart failure. The interim analysis showed significant improvement in the eGFR in the bardoxolone methyl group compared to the eGFR in the placebo group without safety concerns [29]. We must wait for the final analysis report.

## 3. Hypoxia-Inducible Factor (HIF)

### 3.1. HIF–HRE Pathway

**Figure 3:**
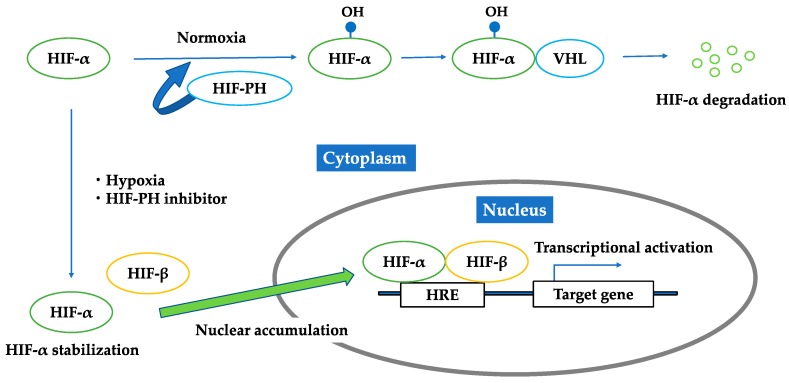
Regulation of HIF under normoxic condition; hypoxic condition; HIF-PH inhibitor. Under normoxic conditions, HIF-α is hydroxylated by HIF-PH and then recognized by von Hippel–Lindau tumor suppressor protein to be degraded. Under hypoxia or the existence of HIF-PH inhibitor, HIF-α is not hydroxylated, but is stabilized in cytoplasm and forms a heterodimer with HIF-β. This heterodimer translocates into the nucleus, binds to the hypoxia responsive elements, and activates downstream genes.

Growing evidence has shown that chronic hypoxia in the tubulointerstitium is a final common pathway to CKD [7,38,39]. Hypoxia is caused by various factors, such as a loss of peritubular capillaries, reduction in peritubular capillary flow, fibrosis of the tubulointerstitium, oxygen demand of resident kidney cells, oxidative stress, and renal anemia. Hypoxia-inducible factor (HIF) is a key modulator of the transcriptional response to hypoxic stress [40,41]. HIF is a heterodimer with oxygen-regulated α-subunits (HIF-1α, 2α, 3α) and a consistently present β-subunit. The three isoforms of the α-subunit combine with the β-subunit in the nucleus and bind to DNA sequences called hypoxia response elements (HRE) to regulate expression of different target genes. There are more than 100 target genes of HIF that are mainly involved in angiogenesis, erythropoiesis, glycolysis, cell differentiation, and apoptosis [42], which play protective and pathogenic roles in ischemic injury. HIF-2α is known as the main regulator of erythropoietin production [43]. HIF is continuously produced, and its production is regulated by the rate of its destruction. Under normoxic conditions, HIF α-subunits are hydroxylated by HIF-prolyl hydroxylase (HIF-PH), known as prolyl hydroxylase domain (PHD) enzymes, and then recognized by von Hippel–Lindau tumor suppressor protein (pVHL), which functions as an E3 ubiquitin ligase to be degraded. In addition, during hypoxia, the HIF α-subunits escape from the oxygen-dependent hydroxylation of PHD enzymes (Figure 3). PHD enzymes require oxygen as a substrate, and thus, they work as the oxygen sensor regulating HIF [44]. HIF stabilization by HIF-PH inhibitors would promote endogenous erythropoietin production in CKD patients with renal anemia and may work beneficially to renal protection [45].

### 3.2. HIF-PH Inhibitor as an HIF Stabilizer

**Figure 4:**
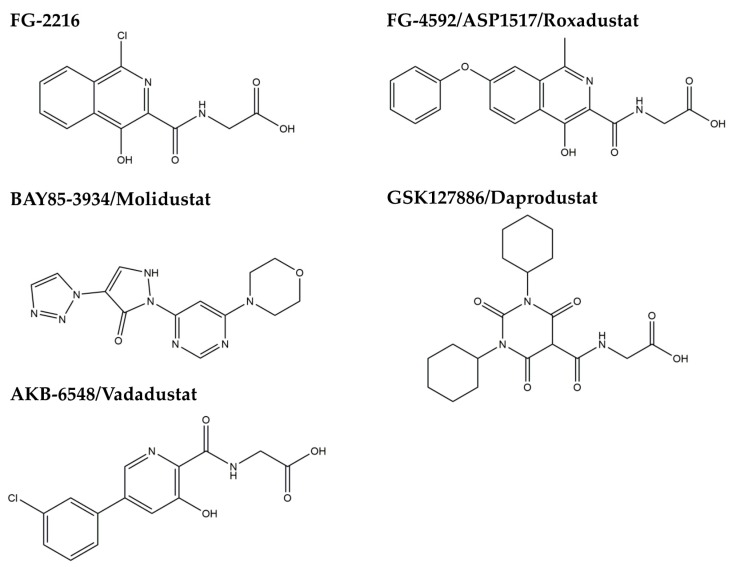
Chemical structures of HIF-PH inhibitors.

Accumulating evidence shows that HIF might be functionally inhibited in the CKD state. There are reports showing that oxidative stress impairs HIF transcriptional activity in diabetic nephropathy [46,47] and that a uremic toxin, indoxyl sulfate, may impair HIF function [48]. Because HIF degradation is dependent on HIF-prolyl hydroxylases (HIF-PH), they have been a good therapeutic target to stabilize HIF. HIF-PH are nonheme iron-containing dioxygenases that require oxygen and 2-oxoglutarate as cosubstrates and iron and ascorbate as cofactors for their enzymatic activity [45]. Cobalt, a chelator of iron, has been reported to improve kidney injury in several CKD animal models [49,50,51]. However, cobalt could not be used in humans because of adverse effects, such as respiratory irritation, cardiomyopathy, and carcinogenesis. Therefore, a new class of HIF-PH inhibitors are under development. At least six HIF-PH inhibitors are now under clinical trials [52], and the results of phase 3 trials will be available within two to three years. Though they have been primarily developed for the treatment of renal anemia, the pleiotropic effects of HIF activation are expected to be beneficial to renal protection (Figure 4).

### 3.3. Brief Summary of Targeting HIF

Because HIF is the master regulator of hypoxic adaptation responses, it is a good therapeutic target for hypoxia tolerance in the kidney, and HIF-PH inhibitors seem to be promising agents. On the other hand, there are reports that inappropriate activation of HIF may lead to kidney fibrosis [53,54] and tumorigenesis [55,56], probably owing to the pleiotropic effects of HIF. We must wait for the clinical trial results and carefully analyze its side effects and hard end points, including mortality and the rate of cardiovascular events.

## 4. Kruppel-Like Factor 4 (KLF4)

### 4.1. KLF4 Modulates Podocyte Phenotype and Attenuates Proteinuria

Kruppel-like factors (KLFs) are zinc finger transcription factors involved in various cellular processes, such as cell differentiation, apoptosis, and cell proliferation [57]. Previous reports have shown the importance of KLFs in glomerular disease. KLF15 has been reported as a key regulator of podocyte differentiation, which mediates retinoic acid- or glucocorticoid-induced restoration of the gene expression of podocyte differentiation markers [58,59]. Additionally, KLF6 has been determined to be an essential regulator of mitochondrial function in podocytes [60].

**Figure 5:**
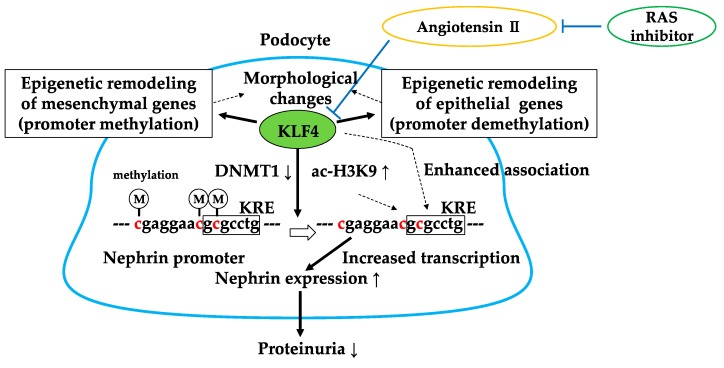
Gene-selective epigenetic regulation via KLF4. KLF4 regulates podocyte phenotype and function through decreased binding of DNMT1 and acetylation of H3K9 in the nephrin promoter region. Angiotensin II-mediated reduction of KLF4 is inhibited by RAS inhibitors. DNMT1↓: decreased binding of DNMT1 in the nephrin promoter, ac-H3K9↑: increased acetylation of H3K9 in the nephrin promoter. KRE: KLF4 response element.

We have recently found that KLF4 is highly expressed in glomerular podocytes, and its expression is decreased in proteinuric status in both animal models and humans [61]. KLF4 is mainly expressed in epithelial cells of organs such as the gastrointestinal tract, skin, lungs, and testes [62,63,64]. It has the ability, along with other factors (OCT4, SOX2, and c-MYC), to reprogram somatic cells into induced pluripotent stem (iPS) cells [65,66,67,68] by activating the expression of epithelial genes during the initial phase of reprogramming. We examined the expression of KLF4 in the adriamycin (ADM) nephropathy model, the puromycin aminonucleoside nephropathy model, and the db/db diabetic nephropathy model, and a decrease in KLF4 expression was observed in all models. Similarly, the immunofluorescence staining on renal biopsy samples from patients with minimal change disease, focal segmental glomerulosclerosis, and diabetic nephropathy showed decreased glomerular expression of KLF4. Transient restoration of KLF4 expression in podocytes in the ADM nephropathy model either by the hydrodynamic-based gene transfer method [69] or transgenic expression resulted in a sustained increase in nephrin expression and a decrease in albuminuria. In addition, podocyte-specific KLF4-knockout mice were more susceptible to ADM nephropathy. KLF4 overexpression in cultured human podocytes increased the expression of nephrin and other epithelial markers and reduced mesenchymal gene expression. DNA methylation profiling and bisulfite genomic sequencing revealed that KLF4 expression reduced methylation at the nephrin promoter and the promoters of other epithelial markers; in contrast, methylation was increased at the promoters of genes encoding mesenchymal markers, suggesting selective epigenetic regulation of podocyte gene expression via KLF4. Moreover, we examined chromatin immunoprecipitation (ChIP) analysis to find out the mechanisms of KLF4-induced changes in nephrin expression. The results revealed that DNA methyltransferase 1 (DNMT1) bound to the nephrin promoter region and was significantly reduced in KLF4-overexpressing podocytes. Additionally, KLF4 overexpression significantly increased acetylated histone protein acetyl-H3K9 associated with the nephrin promoter region containing the KLF4 response element (KRE). These results suggest that KLF4 modulates podocyte phenotype and attenuates proteinuria through epigenetic regulation (Figure 5).

### 4.2. RAS Inhibitor Attenuates Proteinuria in Part via KLF4

RAS inhibitors are widely used in the treatment of CKD and hypertension. The antiproteinuric and renoprotective effect of RAS inhibition was conventionally explained by a reduction in glomerular hydrostatic pressure through dilation of the afferent and efferent arterioles [70]. Recently, trophic effects of RAS inhibition such as reduction of oxidative stress, inflammation, and preservation of the podocyte slit diaphragm structure have been noticed, but its precise mechanism remains unclear.

**Figure 6:**
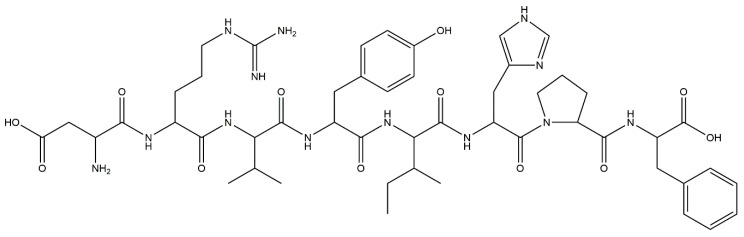
The chemical structure of angiotensin II.

We have recently reported that treatment with an ARB reduced methylation of the nephrin promoter in an ADM nephropathy model with recovery of KLF4 expression and a decrease in albuminuria [71]. Furthermore, the effect of ARB on albuminuria and nephrin promoter methylation was attenuated in podocyte-specific KLF4-knockout mice. In cultured human podocytes, angiotensin II (Figure 6) reduced KLF4 expression and caused methylation of the nephrin promoter with decreased nephrin expression. ChIP analysis revealed that angiotensin II treatment caused decreased acetylation of H3K9 and increased binding of DNMT1 in the nephrin promoter, which were inhibited by ARB. In patients with proteinuric kidney diseases, methylation-specific PCR analysis using microdissected glomeruli showed increased methylation at the nephrin promoter with decreased KLF4 and nephrin expression. KLF4 expression in ARB-treated patients was higher than in patients without ARB treatment, as confirmed by immunofluorescent staining. These results show that angiotensin II can modulate epigenetic regulation in podocytes and that RAS blockade can exert therapeutic effects through epigenetic modulation via KLF4 (Figure 5).

### 4.3. Brief Summary of Targeting KLF4

KLF4 regulates podocyte phenotype through gene-selective epigenetic control, and the antiproteinuric effects of RAS inhibition could be partly explained by epigenetic modulation via KLF4 in glomerular podocytes [71]. Likewise, in generating iPS cells with the appropriate combination of transcription factors, treatment with the appropriate transcription factors such as KLF4 may be enough to change or reset the epigenetic status in disease states. Epigenetic modulation through transcription factors involved at specific genes and tissues may be beneficial in reducing side effects compared to systemic administration of direct epigenetic modulators such as histone modifiers or DNA methyltransferases. Furthermore, since KLF4 is expressed in epithelial cells including tubular epithelium, the role of KLF4 in renal fibrosis is also featured. In unilateral ureteral obstruction (UUO) mice as a model of renal interstitial fibrosis, a decrease in KLF4 expression was observed, indicating that KLF4 has antifibrotic action in the kidney [72,73]. Moreover, it is implied that KLF4 may reduce inflammation stimulated by TGF-β1 in cases of renal fibrosis caused by diabetic nephropathy [74]. These results suggest that KLF4 may be a potential therapeutic target for kidney disease.

## 5. Conclusions

The present review focused on the transcription factors that seem to be promising for the treatment of CKD. Agents targeting Nrf2 and HIF are now under clinical trial, and RAS inhibitors targeting KLF4 in part have been used in daily practice. The three transcription factors mentioned in this review are especially important because agents targeting them are almost in clinical use or have actually already been used. Transcription factors often regulate multiple target genes; therefore, their pleiotropic effects may sometimes be harmful. To reduce the side effects caused by systemic modulation of transcription factors, new approaches are expected. One is to reveal the organ-, tissue-, and cell type-specific induction pathways of transcription factors as a therapeutic target. Another way is to develop a drug delivery system to the specific target cells. Investigation of more efficient and specific therapies targeting transcription factors is necessary in future work.
